# IL-2 Induces Transient Arrest in the G1 Phase to Protect Cervical Cancer Cells from Entering Apoptosis

**DOI:** 10.1155/2019/7475295

**Published:** 2019-09-26

**Authors:** María del Carmen Lagunas-Cruz, Arturo Valle-Mendiola, Jonathan Trejo-Huerta, Leticia Rocha-Zavaleta, María de Lourdes Mora-García, Adriana Gutiérrez-Hoya, Benny Weiss-Steider, Isabel Soto-Cruz

**Affiliations:** ^1^Molecular Oncology Laboratory, Cell Differentiation and Cancer Research Unit, FES Zaragoza, National University of Mexico, Batalla 5 de Mayo S/n Col. Ejército de Oriente, CP 09230 Mexico City, Mexico; ^2^Department of Molecular Biology and Biotechnology, Institute of Biomedical Research, National University of Mexico, Mexico City, Mexico; ^3^Cátedra CONACYT, CONACyT, Avenida Insurgentes Sur 1582, Col. Crédito Constructor Del. Benito Juárez, 03940 Mexico City, Mexico

## Abstract

Interleukin 2 (IL-2) has been used for the treatment of different types of cancer that express the IL-2 receptor (IL-2R). However, the effect of IL-2 on cervical cancer cells is unknown. IL-2R is present in normal cells of the immune system but not in the healthy cervix. We report that IL-2R is expressed in cervical cancer cells. IL-2 decreases cervical cancer cell proliferation via transient arrest of the G1 phase, which does not result in apoptosis or senescence. IL-2 upregulates the expression of p53 and p21 and downregulates cyclin D. In addition, we report the resistance of cervical cancer cells to treatments that induce apoptosis in HeLa and INBL cells. When arrested cells were treated with cisplatin, the cytokine protected cells from apoptosis induced by cisplatin. The effects of IL-2 on the cell cycle do not induce cellular senescence or activate the proapoptotic protein Bax. The cell arrest induced by IL-2 is conferring protection to cells against apoptosis.

## 1. Introduction

Cervical cancer is the third most frequent type of cancer in women around the world with a global incidence of 500,000 and mortality of 250,000 [1]. In the past thirty years, significant advances lead to our understanding of the initiation process and development of cervical carcinogenesis [2]. Current radical surgery, radiation, and chemotherapy can cure more than 85% of women with cervical cancer in early stages [3]. However, in stage IVB, recurring or persistent cervical cancer does not respond to these typical treatments and remains a significant cause of death related to cancer [4]. Thus, it is necessary to develop efficient treatments for this type of cancer.

Interleukin 2 (IL-2) has been used to treat diverse types of cancer that express the IL-2 receptor (IL-2R) such as intestinal cancer [5, 6], esophageal cancer [7], and head and neck cancer [8]. Normal cervical cells do not express IL-2R, but the expression of IL-2R in cervical cancer cells has been reported by some groups and by our research group [9, 10]. On the other hand, IL-2R is present in normal cells of the immune system, for example, lymphocytes [11], natural killer cells [12], and dendritic cells [13]. Our workgroup reported that treatment with 100 IU of IL-2 induces a decrease in the phosphorylation of JAK3 and STAT5 proteins involved in the proliferation of cervical cancer cells [14]. JAK3 and STAT5 are proteins that have been reported to be involved in the regulation of cell proliferation [15].

Most chemotherapeutic drugs govern the growth of cancer cells by inducing an arrest at either the G1/S or the G2/M phase. Cells induce an arrest at cell-cycle checkpoints for a short time to allow for cellular-damage repair [16]. Checkpoint signalling may also induce the activation of pathways ending in apoptosis if cellular damage fails to repair correctly [17]. Irregularities in cell-cycle checkpoints may result in gene mutations, chromosome damage, and aneuploidy that can contribute to tumorigenesis [18]. Of the cell-cycle transcriptional influx, G1-S transcription is well characterised because of its fundamental role in the tightened regulation of the G1 to S phase transition [19]. The molecules that regulate cell-cycle progression are well described. One of the critical proteins is p53, a key tumour suppressor, a strong apoptosis-inducer, and a prognostic marker in cancer. Approximately 50% of human tumours bear a mutation in the p53 gene [20]. This nuclear transcription factor accumulates in response to cellular stress, together with DNA damage and oncogene activation, and triggers the transcriptional activation of p21 and Bax, leading to cell-cycle arrest, senescence, or apoptosis [19, 21–24]. The acetylation of lysine 382 is crucial for p53 activation since this modification regulates the promoter-specific activation of p53 target genes to respond to various stress signals [25]. p53 inhibits cell-cycle progression at the G1 or G2/M phase via induction of the cell-cycle inhibitor p21 (also known as CDKN1A, WAF1, or CIP1) [26].

Cisplatin is a well-known antitumour drug and remains a best-selling anticancer drug worldwide [27]. The antitumour activity of cisplatin derives from its capacity to form bifunctional DNA cross-links. The main adducts formed by cisplatin with DNA are guanine-guanine (GG) or adenine-guanine (AG) intrastrand cross-links via the coordination of Pt to N7 of guanine inhibiting DNA synthesis and mitosis, and activating apoptotic cell death [28]. Cisplatin has been largely used to cure distinct types of cancer such as head and neck [29], lung [30], ovarian [31], leukaemia [32], breast [33], brain [34], kidney [35], testicular [36], and cervical cancer [37]. In general, cisplatin and other platinum-related compounds are effective anticancer drugs that kill cancer cells by inducing DNA damage, inhibiting DNA synthesis and mitosis, and activating apoptotic cell death [27].

IL-2 promotes the proliferation of normal B and T cells [38]. Nevertheless, our workgroup reported that treatment with 100 IU of IL-2 induces a decrease in the phosphorylation of proteins involved in the proliferation of cervical cancer cells, which potentially causes a reduction in cell proliferation [39]. However, in cervical cancer cells, the mechanism that IL-2 activates to induce a decrease in cell proliferation is unknown.

Considering that IL-2 leads to a reduction in cell proliferation and possibly apoptosis, the current investigation was undertaken to evaluate the effect of IL-2 on the inhibition of proliferation of cervical cancer cells and to determine if this inhibition is due to an induction of cell death. For this purpose, we analysed the exposure of phosphatidylserine on the surface membrane, the fragmentation of DNA, and the expression of Bax protein involved in apoptosis regulation. Also, we analysed the expression of the cell-cycle proteins p53, p21, and cyclin D. Furthermore, we evaluated the effect of IL-2 on the induction of cell senescence. Finally, we assessed the apoptosis induced by cisplatin in arrested cervical cancer cells. Here, we show that IL-2 induces a transient arrest in the INBL cell line. The observed cell-cycle arrest did not cause the activation of cellular senescence, and only a small proportion of cells underwent apoptosis. IL-2 does not activate the proapoptotic protein Bax. Surprisingly, treatment with IL-2 protects the cervical cancer cell line INBL from entering apoptosis induced by cisplatin.

## 2. Materials and Methods

### 2.1. Biological Material

The HeLa cell line, derived from an adenocarcinoma, was purchased from the American Type Culture Collection (ATCC-CCL-2). INBL, an HPV 18 cell line derived from an invasive stage IVB squamous cell carcinoma, was established at the Cell Differentiation Laboratory of FES Zaragoza [40]. Both cell lines were grown in RPMI-1640 medium (Microlab, Mexico) supplemented with 5% foetal bovine serum (FBS; Invitrogen, USA). All cell cultures were maintained in an incubator at 37°C, 5% CO_2_, and saturated humidity.

### 2.2. Antibodies

In this study, the following antibodies were used: IL-2R*α* PE mouse monoclonal antihuman IL-2Ra CD25 PE, clone M-A251 (Catalog 555432, BD, USA); IL-2R*β* CD122 FITC mouse monoclonal antibody, clone TU27 (Catalog 11-1228-42, Thermo Fisher Scientific, USA); IL-2R*γ* CD98 mouse monoclonal antibody, clone E-5 (Catalog sc-376815, Santa Cruz Biotechnology, USA); goat anti-mouse IgG-FITC secondary antibody (Sigma Aldrich); isotype control; mouse monoclonal antihuman IgG3-FITC antibody, clone HP-6050 (Catalog F4641, Sigma Aldrich); and PE mouse IgG1, *κ* isotype control, clone MOPC-31C (Catalog 555749, BD Pharmingen).

### 2.3. Detection of IL-2 Receptor Subunits

Approximately 3 × 10^5^ HeLa and INBL (positive control) cervical cancer cells per condition were seeded onto Petri dishes and grown in the presence of 10 IU/ml of IL-2 overnight. Two cell cultures without IL-2 were used, one as a baseline control and the other as an autofluorescence control (AFC). Cells were harvested and rinsed with 1 ml of phosphate-buffered saline (PBS). Cells were fixed with 2% paraformaldehyde for 20 minutes. For permeabilisation, cells were fixed with 2% paraformaldehyde, rinsed with PBS, and incubated with cold methanol (JT Baker, USA) for 20 minutes. An anti-alpha, anti-beta, or anti-gamma IL-2R subunit primary antibody (1 : 100) was added, and the cells were incubated for 1.5 hours at 4°C while shaking. Isotype controls were used to measure the level of nonspecific background signal caused by primary antibodies. The cells were incubated with isotype control antibodies (1 : 100) for 1.5 hours at 4°C. Cells were then washed, and a FITC-conjugated secondary antibody (Santa Cruz Biotechnology, USA) was added when necessary; the mixture was incubated for 45 minutes. Proteins were detected by flow cytometry on a FACSAria II cytometer (BD, USA). Data were examined using Flowing Software version 2.5.1.

### 2.4. Cell Proliferation Assay

Approximately 2 × 10^3^ HeLa and INBL cervical cancer cells were seeded on 96-well plates and incubated in the presence of 10, 50, and 100 IU/ml IL-2 for 24 and 48 hours at 37°C. The negative control was generated by incubating cells for 24 and 48 hours without treatment. After each time point, the medium was discarded, and cells were fixed with 1.1% glutaraldehyde for 20 minutes (Sigma, USA). Subsequently, cell samples were stained with 0.1% violet crystal (Sigma, USA), which was incorporated into nucleic acids. Finally, 10% acetic acid was added, and the absorbance was read on a microplate reader (BioRad, USA) at 570 nm.

### 2.5. Annexin V-PE Assay

Apoptosis was evaluated by annexin-V staining using the Annexin V-PE Apoptosis Detection Kit (BD Pharmingen, USA). In total, 5 × 10^5^ cells were cultured for 24 and 48 hours with the indicated treatments. Cell samples were stained with the Annexin V-PE Apoptosis Detection Kit, following the manufacturer's protocol. Briefly, untreated cells were used to determine and locate the cell population on a cytometer. Cells were treated with 200 *μ*L of ethanol for 20 minutes to induce necrosis. Cells untreated were used to observe cell death under normal conditions, and cells treated with 100 IU/ml of IL-2 were used to analyse apoptosis. Approximately 1 × 10^5^ cells were used for each experimental condition. As a positive control, cells were treated with 2 *μ*g/ml of puromycin (Sigma, USA) for 24 hours to induce apoptosis. The percentage of apoptotic cells was evaluated by flow cytometry using a FACSAria II cytometer. Data were examined using Flowing Software version 2.5.1.

### 2.6. DNA Fragmentation

Approximately 1 × 10^5^ HeLa and INBL cervical cancer cells were grown in the absence or presence of 100 IU/ml of IL-2 for 48 hours. Cells were harvested, rinsed once with PBS, and centrifuged at 327 ×*g* for 5 minutes. The supernatant was discarded, and 1 ml of DNAzol (Thermo Fisher, USA) was added. Cells were resuspended gently by inversion and allowed to stand for 15 minutes at 4°C. Next, 1 ml of absolute ethanol (JT Baker, USA) was added and the mixture was gently stirred by inversion and then centrifuged at 11,752 ×*g* for 12 minutes. The supernatant was eliminated while ensuring not to remove DNA. The pellet was then washed twice with 75% ethanol and centrifuged at 11,752 ×*g* for 5 minutes. Ethanol was removed using a micropipette to avoid losing the pellet. The pellet was allowed to dry at room temperature for 30 minutes and then rehydrated with water free of DNases and RNases. Finally, the products were separated by electrophoresis on a 1.5% agarose gel in a 0.5% TBE in an electrophoresis chamber (Atto Corporation, Japan) at 90 volts for 90 minutes. Bands were observed on a Digi-DOCIT imaging system (UVP, Upland, CA).

### 2.7. RNA Extraction

After 24 and 48 hours of treatment with 100 IU/mL IL-2, cells were washed with PBS, and 1 ml of TRIzol (Thermo Fisher, USA) reagent was added. The homogenized sample was incubated for 10–15 minutes at room temperature. Chloroform (200 *μ*L; Sigma) was added and stirred gently, and the sample was centrifuged at 13792 ×*g* for 15 minutes at 4°C. The aqueous phase was separated, and 500 *μ*L of 100% isopropanol (JT Baker, USA) was added. The sample was then incubated for 10 minutes at room temperature and centrifuged at 13792 ×*g* at 4°C for 15 minutes. The supernatant was removed, washed with 75% ethanol, and centrifuged at 13792*g* for 5 minutes at 4°C. The pellet was dried at room temperature, and 50 *μ*L of water free of RNases was added to dissolve the RNA. RNA content was quantitated by dissolving 1 *μ*L of the sample in 199 *μ*L of RNase-free water, creating a 1 : 200 dilution. The RNA concentration was determined on a biophotometer (Eppendorf) at an absorbance of 260/280 nm.

### 2.8. Retrotranscription (RT-PCR)

cDNA was synthesized from 3 *μ*g of mRNA using 1 *μ*L of RevertAid M-MuLVRT (Thermo Fisher, USA) enzyme, 5 *μ*L of first strand buffer, 4 *μ*L of oligo(dT) 18 primer, 1 *μ*L of 10 mM dNTP mix, and 2 *μ*L of RiboLockRnase inhibitor (20 U/*μ*L). The reaction conditions in the thermocycler were 42°C for 1 hour. To terminate the reaction, samples were heated to 70°C for 5 minutes.

### 2.9. PCR

cDNA was amplified with Taq polymerase (Thermo Fisher, USA) using the primers in Table 1.

Each reaction was prepared with 3 *μ*L of 10x Taq buffer reaction, 2.5 *μ*L of 25 mM MgCl_2_, 0.1 *μ*L of Taq polymerase, 0.5 *μ*L of 10 mM dNTPs, 0.2 *μ*L of the primer of interest, and 1.5 *μ*L of cDNA for a final volume of 20 *μ*L with water free of RNases. The amplification was performed in a thermocycler (PTC-200 Peltier Thermal Cycler ALO28386) as follows: 2 minutes at 94°C, followed by 30 cycles at 94°C for 30 s, 55°C for 30 s, and 72°C for 60 s and 10 minutes at 72°C. The PCR products were separated on a 1.5% agarose gel at 90 to 100 volts.

### 2.10. Activation of p53

Cells (1 × 10^5^) were incubated with 100 IU/ml of IL-2 for 24 and 48 hours to examine the activation of p53. Cells were harvested, washed with PBS, fixed with 2% paraformaldehyde, washed with PBS, and incubated with methanol (JT Baker, USA) for 20 minutes for permeabilisation. Cells were washed twice with 5% FBS in PBS and incubated with an Alexa Fluor 647 mouse anti-p53 (acK382) antibody (BD Biosciences, USA) at a 1 : 100 dilution for 1.5 hours. Cells were analysed by flow cytometry on a FACSAria II cytometer (BD, USA). The results obtained by flow cytometry were analysed using the Flowing Software program version 2.5.1 considering the AFC as 100%, and the percentages of the samples were calculated based on that value.

### 2.11. Cell-Cycle Analysis

We observed a decrease of cell proliferation in HeLa and INBL cell lines at 48 hours; therefore, we decided to determine the phases of the cell cycle at these time points. To determine the cell-cycle phases, 1 × 10^6^ HeLa and INBL cervical cancer cells were seeded and incubated with 100 IU/ml IL-2 for 24 and 48 hours. Cell-cycle determination was performed using the propidium iodide incorporation technique. Cells were fixed using 70% ethanol (JT Baker, USA) for 12 hours, and RNA was removed by incubating with an RNAase (Sigma, USA) for one hour at 37°C. Cells were incubated with 3 *μ*L of propidium iodide (Sigma; 50 mg/mL) for 10 minutes. Cell-cycle phases were determined using flow cytometry on a FACSAria II cytometer (BD, USA). Data were analysed using FCS express 7 De Novo software. To determine if the observed cell arrest is permanent or transient, after 48 hours of cell culture in the presence of IL-2 the medium was removed, and fresh medium without IL-2 was added, leaving the cells in culture for 24 and 48 hours to analyse the phases of the cell cycle.

### 2.12. Senescence Analysis

Senescence was determined using the Senescence Detection Kit (BioVision Incorporate, USA). 2.5 × 10^5^ cervical cancer cells HeLa and INBL were seeded in 12-well plates and incubated with 100 IU/ml of IL-2; cells cultured without serum in the presence of H_2_O_2_ were used as positive control. The peroxide induces the presence of free radicals that activate the senescent process. We used cells cultured in the presence of 10% FBS as negative control. Incubation was carried out for 96 hours. Once the incubation time has elapsed, they were washed with 1 ml of PBS and incubated for 10 to 15 minutes with 500 *μ*L of fixing solution at room temperature. The cells were washed two times with PBS, and 500 *μ*L of staining solution mixture (470 *μ*L of staining solution, 5 *μ*L of staining supplement, and 25 *μ*L of a 20 mg/ml solution of X-gal) was added to each well. Cells were incubated overnight at 37°C and were observed under the microscope to look for the development of blue color.

### 2.13. Cisplatin Treatment on G1-Arrested Cervical Cancer Cells

To determine the amount of cisplatin (Pisa, Mexico) required to induce a decrease in cell population (IC50), a dose-response curve was generated. Approximately 1 × 10^5^ HeLa and INBL cells were seeded onto 60 mm Petri dishes and incubated with and without 1, 2.5, 5, 10, 20, and 30 *μ*g of cisplatin. Apoptosis was detected by staining with annexin-V, using the PE Annexin-V Kit Apoptosis Detection Kit (BD Pharmingen, USA).

To determine the effect of cisplatin on cells arrested in the G1 phase, 1 × 10^5^ HeLa and INBL cells were seeded onto 60 mm Petri dishes and incubated with and without 100 IU/ml of IL-2 for 48 hours. Next, cisplatin (5 *μ*g/ml) was added, and the cells were incubated for 24 and 48 hours. Apoptosis was detected by staining with annexin-V, using the PE Annexin-V Apoptosis Detection kit (BD Pharmingen, USA). Cells were detached and stained with the PE Annexin V Apoptosis Detection Kit, following the manufacturer's protocol. The percentage of apoptotic cells was evaluated by flow cytometry using a FACSAria II cytometer from BD. Data were analysed using Flowing Software 2.5.1.

### 2.14. Statistical Analysis

All data were obtained from three independent experiments for statistical analysis. We used the two-way repeated measures ANOVA and Student's *t*-test of paired, nonparametric data with GraphPad Prism 5.0 statistical package.

## 3. Results

### 3.1. Presence of the IL-2 Receptor in HeLa and INBL Cervical Cancer Cell Lines

**Figure 1:**
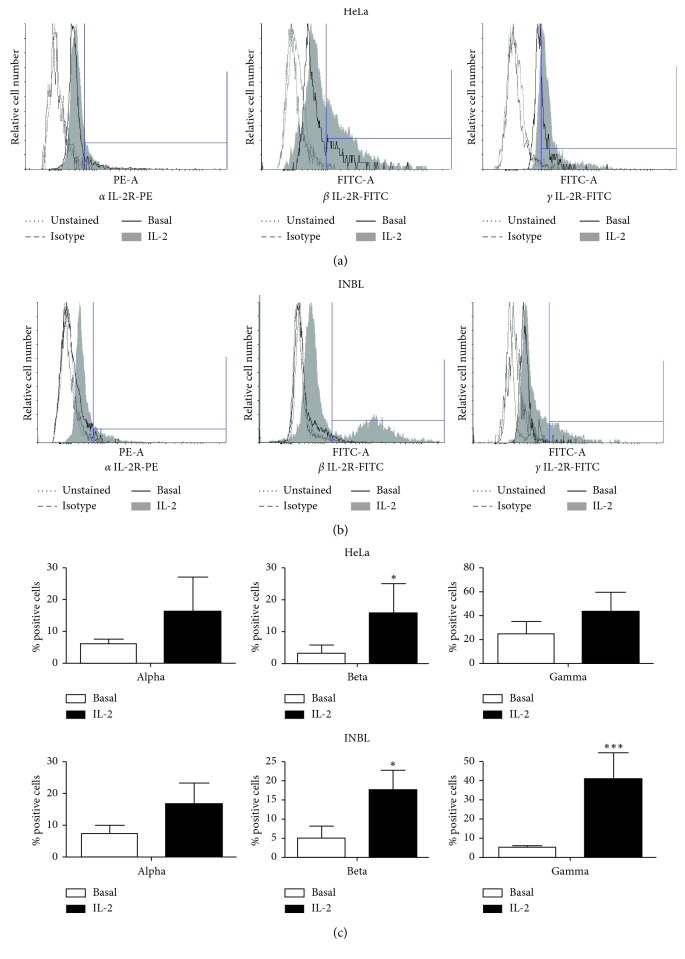
Presence of the alpha, beta, and gamma subunits (IL-2R) in the cervical cancer cell lines HeLa and INBL. The expression of the IL-2R subunits was determined using specific antibodies for the alpha, beta, and gamma subunits by flow cytometry on a FACSAria II cytometer (BD, USA). The histograms corresponding to the HeLa cell line (a) and the INBL cell line (b) are shown. We defined the gate for positive events such that all negative controls (isotypes) represented 1% of events. Then, this gate was applied to all the conditions. Representative flow cytometry plots of one out of three independent experiments. (c) The graph representation of the basal expression compared to the expression after the stimulation with IL-2 is shown. The expression of the IL-2R subunits is statistically significant. ^*∗*^*P* < 0.05, ^*∗∗∗*^*P* < 0.001. Results are the median of three independent experiments.

To evaluate whether HeLa and INBL cells respond to IL-2 treatment, we determined the presence of the IL-2R (alpha, beta, and gamma subunits) on the cell membrane using specific antibodies. The results revealed the expression of all three subunits of the IL-2 receptor in both cell lines (Figure 1). Cells were grown in the presence of IL-2 to determine whether the subunits are inducible.  Figure 1(a) shows the results for HeLa cells, and Figure 1(b) shows the results obtained for INBL cells. In each histogram, the bridged line represents the isotype to measure the level of nonspecific background signal caused by primary antibodies, and the grey area represents cells with treatment. The alpha subunit of IL-2R is inducible with IL-2 treatment in both cell lines. The basal expression of the alpha subunit in HeLa cells increases from 6.07% to 16.36% compared with expression in INBL cells that increase from 7.32% to 16.8% (Figure 1(c)). The beta expression increases from 3.24% to 15.87% in HeLa cells and from 5.08% to 17.69% in INBL cells (Figure 1(c)). The gamma expression rises from 24.61% to 41.03% in HeLa cells (Figure 1(c)), and in INBL cells, an increase from 4.48% to 34.25% is observed (Figure 1(c)). The graph representation is shown in Figure 1(c) for HeLa and INBL cells.

### 3.2. IL-2 Inhibits Cervical Cancer Cell Growth

**Figure 2:**
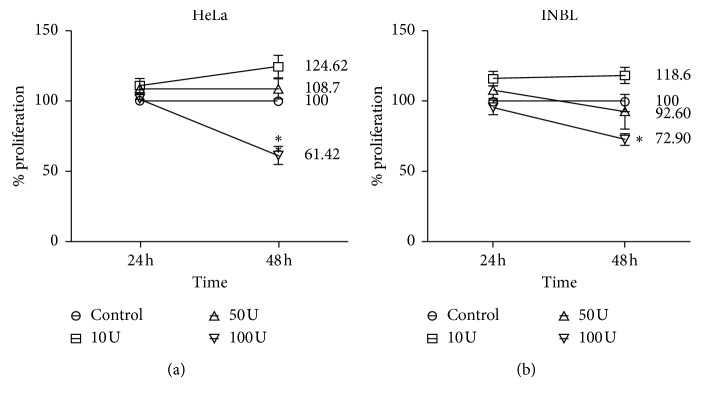
IL-2 induces a decrease in cell proliferation. Cells were incubated in the presence or absence of 10, 50, and 100 IU/ml of IL-2 for 24 and 48 hours. (a) In the HeLa cell line, a significant decrease in cell proliferation was observed after 48 hours of incubation with 100 IU/ml of IL-2. (b) In the INBL cell line, there is a significant decrease in cell proliferation at 48 hours of incubation with 100 IU/ml of IL-2. ^*∗*^*P* < 0.05. Results are the median of five independent experiments.

We evaluated the optimal concentration of IL-2 required to inhibit the proliferation of tumour cells. In normal serum, IL-2 is synthesized physiologically at 7.46 ± 2.98 IU/mL levels [41], but we have demonstrated that 100 IU/ml of IL-2 is necessary for the activation of the JAK/STAT pathway and the proliferation in normal lymphocytes [14]. To determine the effect of different concentrations of IL-2 on cervical cancer cell proliferation, HeLa and INBL cells were grown in the presence of 10, 50, and 100 IU/ml of IL-2 for 24 and 48 hours (Figure 2). For the HeLa and INBL cell lines, we observed an increase in cell proliferation with 10 IU and 50 IU of IL-2. On the contrary, for HeLa cells, we observed a 38.6% of inhibition at 48 hours with 100 IU/mL of IL-2 compared with the control (Figure 2(a)). For the INBL cell line, 31.1% of inhibition occurred at 48 hours (Figure 2(b)). These results demonstrate that 100 IU/ml of IL-2 is the optimal concentration to inhibit cell growth since it decreases the proliferation of HeLa and INBL cells at 48 hours. These data are consistent with the results shown by our group that low doses of IL-2 induce phosphorylation of JAK3 and STAT5 and an increase in cell proliferation, while high doses of IL-2 have opposite effects since they cause a reduction in JAK3 and STAT5 phosphorylation [39]. Once the optimal concentration of IL-2 was determined to decrease cell proliferation, we used 100 IU/ml of IL-2 for the following experiments.

### 3.3. IL-2 Does Not Induce Apoptosis in HeLa or INBL Cells

**Figure 3:**
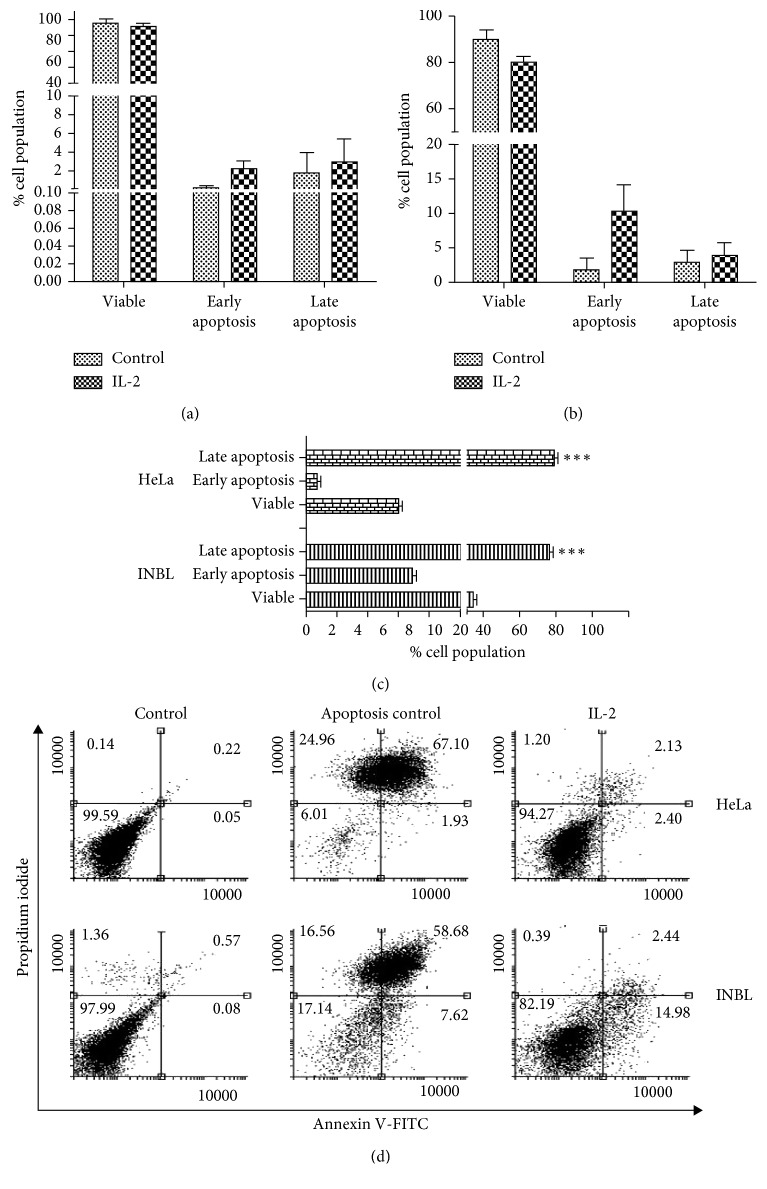
Effect of IL-2 on cervical cancer cell apoptosis. HeLa (a) and INBL (b) cells were incubated in the presence or absence of IL-2 for 48 hours. Cells were fixed and incubated in the presence of annexin-V and evaluated by flow cytometry. No significant apoptosis was observed in either cell line. Puromycin-induced apoptosis was used as a positive control (c). Dot plots show apoptosis percentage after propidium iodide and FITC-Annexin V flow cytometry analysis on a FACSAria II cytometer (d). Representative flow cytometry plots of one out of three independent apoptosis assays.

Considering that IL-2 induces a decrease in cell proliferation, we analysed whether IL-2 activates the process of apoptosis. We used three techniques that determine different phases of apoptosis. First, we used annexin-V to determine the exposure of phosphatidylserine in the cell membrane (Figure 3). Treatment of HeLa cells with 100 IU/ml of IL-2 (Figure 3(a)) induced 2.3% early apoptosis and 3.05% late apoptosis compared with the control. For the INBL cell line (Figure 3(b)), IL-2 induced 10.10% early apoptosis and 3.97% late apoptosis compared with the control.  Figure 3(c) shows the positive controls for apoptosis of both HeLa and INBL cell lines, and Figure 3(d) shows the flow cytometry plots of the apoptosis assay.

**Figure 4:**
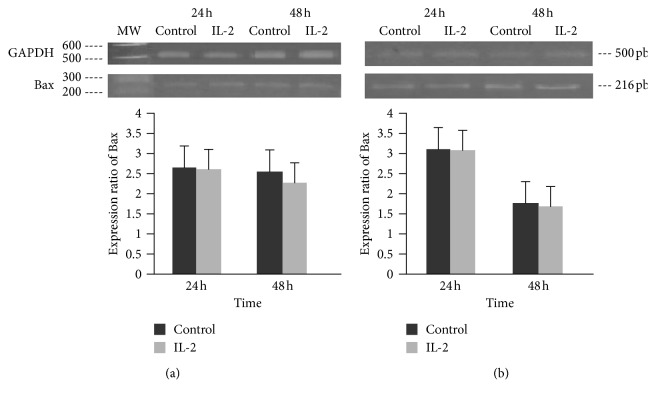
Effect of IL-2 on the expression of Bax. HeLa and INBL cells were incubated with or without 100 IU/ml of IL-2 for 24 and 48 hours. RNA was obtained, and RT-PCR was performed. The expression ratio of Bax in HeLa cells (a) and INBL cells (b) is shown. Statistical analysis was performed with a Student's *t-*test of paired nonparametric data. It was performed using the Statistical Package Graphpad Prism 5.0. ^*∗*^*P* < 0.05.

To further demonstrate that IL-2 did not induce apoptosis, we determined the relative expression of the proapoptotic Bax protein at 24 and 48 hours. The Bax protein did not show any increase compared with the negative control (Figures 4(a) and 4(b)). One process associated with apoptosis is the degradation of DNA in a specific manner between nucleosomes. Therefore, HeLa and INBL cells were grown in the presence of IL-2 to test whether IL-2 treatment induces DNA fragmentation. The DNA was not damaged since the ladder pattern was not present in either cell lines. This absence of DNA degradation contrasts with the death by apoptosis revealed in the fragmented DNA of cells treated with puromycin (Supplementary [Supplementary-material supplementary-material-1]).

### 3.4. IL-2 Induces a Transient Arrest of the Cell Cycle in Cervical Cancer Cells

Since we demonstrated that the decrease in cell proliferation was not by inducing apoptosis, we analysed the phases of the cell cycle. Cell-cycle control ensures the fidelity of DNA replication and cell division to prevent the erroneous transmittal of genetic information. Two main checkpoints located in the transition between G1/S and G2/M regulate the cell cycle. In cancer cell lines, circumventing control checkpoints is one of the most important strategies. HeLa and INBL cells were incubated with 100 IU/ml of IL-2 for 24 and 48 hours to evaluate the effect of IL-2 on the cell cycle. The results were compared with those of a control group formed by the same type of cells without treatment. DNA distribution was determined during each phase of the cell cycle.

**Figure 5:**
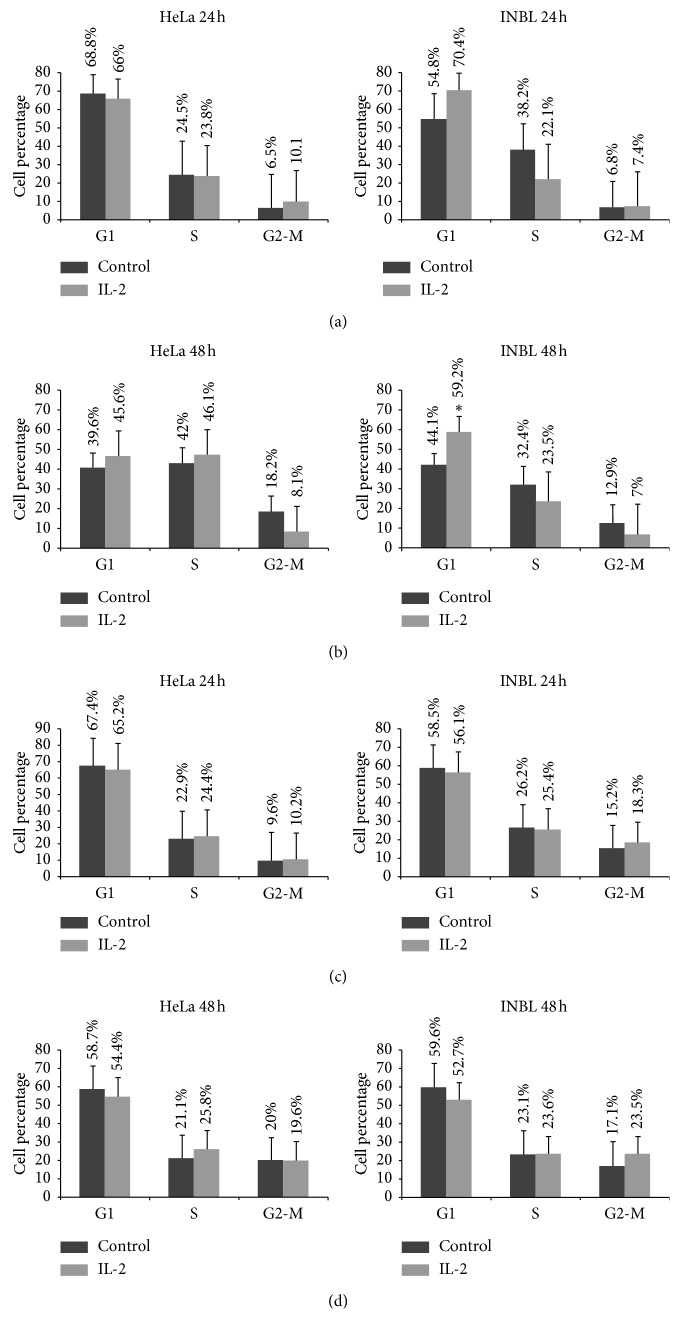
Effect of IL-2 on cell-cycle phases. HeLa and INBL cells were incubated with and without 100 IU/ml of IL-2 for 48 hours to determine cell-cycle phases. (a, b) A significant arrest in G1 phase was observed after IL-2 treatment for 48 hours in INBL cell line. After inducing G1 arrest by treating cells with 100 IU of IL-2 for 48 hours, the medium containing IL-2 was removed, and fresh culture medium supplemented with 10% FBS was added. The cells were cultured further for 24 and 48 hrs. (c, d) HeLa and INBL cells reentry to the cell cycle. ^*∗*^*P* < 0.05.

The results showed a nonsignificant increase in HeLa cells in the G1 phase at 24 hours (Figure 5(a)). In INBL cells, the G1 phase of the cell cycle increases from 54.8% to 70.4% at 24 hours. At 48 hours in HeLa cells, phases G1 and S showed a nonsignificant increase from 39.6% to 45.6% and from 42.0% to 46.1%, respectively (Figure 5(a)). On the contrary, in INBL cells, we observed a significant increase in phase G1 from 44.1 to 59.2% (Figure 5(b)). When we determined that there was an arrest in the G1 phase of the cell cycle and that there was a decrease of the G1 arrest after 48 hours, the medium with IL-2 was removed and fresh medium supplemented with 10% FBS was added to determine whether the cells remained arrested or resumed their proliferation. Both results were compared with cells without treatment, cultured for 24 hours. The results demonstrate that cells resume their proliferation and enter the cell cycle after 24 hours in culture with media supplemented with bovine serum (Figures 5(b) and 5(c)). In Supplementary [Supplementary-material supplementary-material-1], the cell-cycle plots from the analysis of the cytometry data are shown.

**Figure 6:**
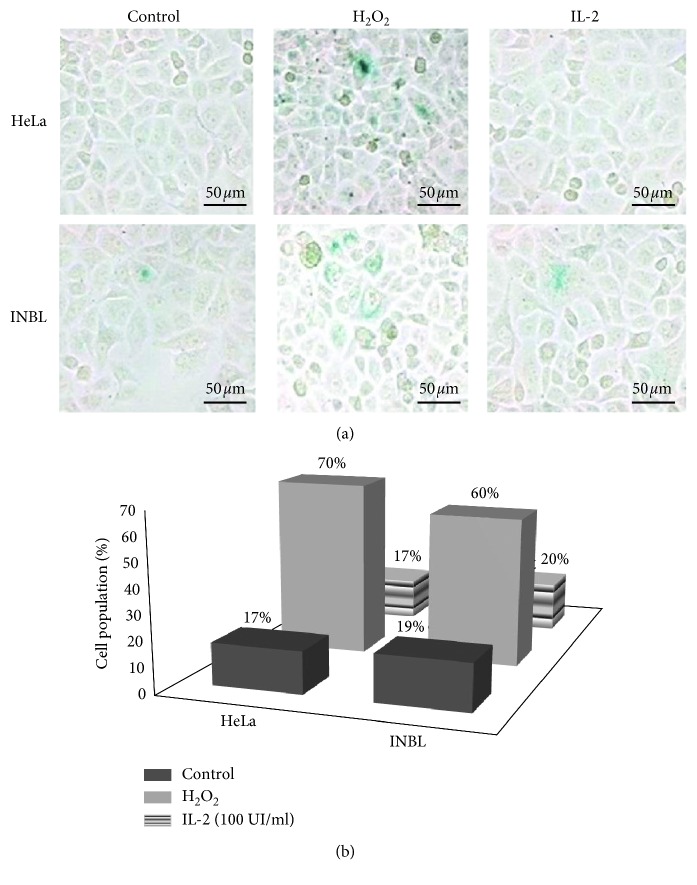
IL-2 does not induce senescence in HeLa and INBL cells. HeLa and INBL cells were treated with 100 IU/ml of IL-2 for 96 hours, and beta-galactosidase activity characteristic of senescent cells was analysed (blue color) using a commercial senescence detection kit. A representative image of three independent experiments is shown.

These results indicate that treatment with 100 IU/ml of IL-2 induces a transient cycle arrest in the G1 phase at 48 hours in INBL cells compared with the control. Therefore, we analysed whether this arrest ended in senescence. Cellular senescence constitutes an alternative route of response to programmed cell death and is of vital importance to suppressing the formation of cancer cells. However, no senescence activation was observed in either HeLa or INBL cells (Figure 6).

### 3.5. Effect of IL-2 on the Expression and Activation of Cell-Cycle-Related Proteins in Cervical Cancer Cell Lines

To further explore the molecular changes related to the G1 phase arrest, we undertook the task of identifying possible targets of IL-2 that may connect with the transition from the G1 to S phases, such as p53, p21, and cyclin D.

**Figure 7:**
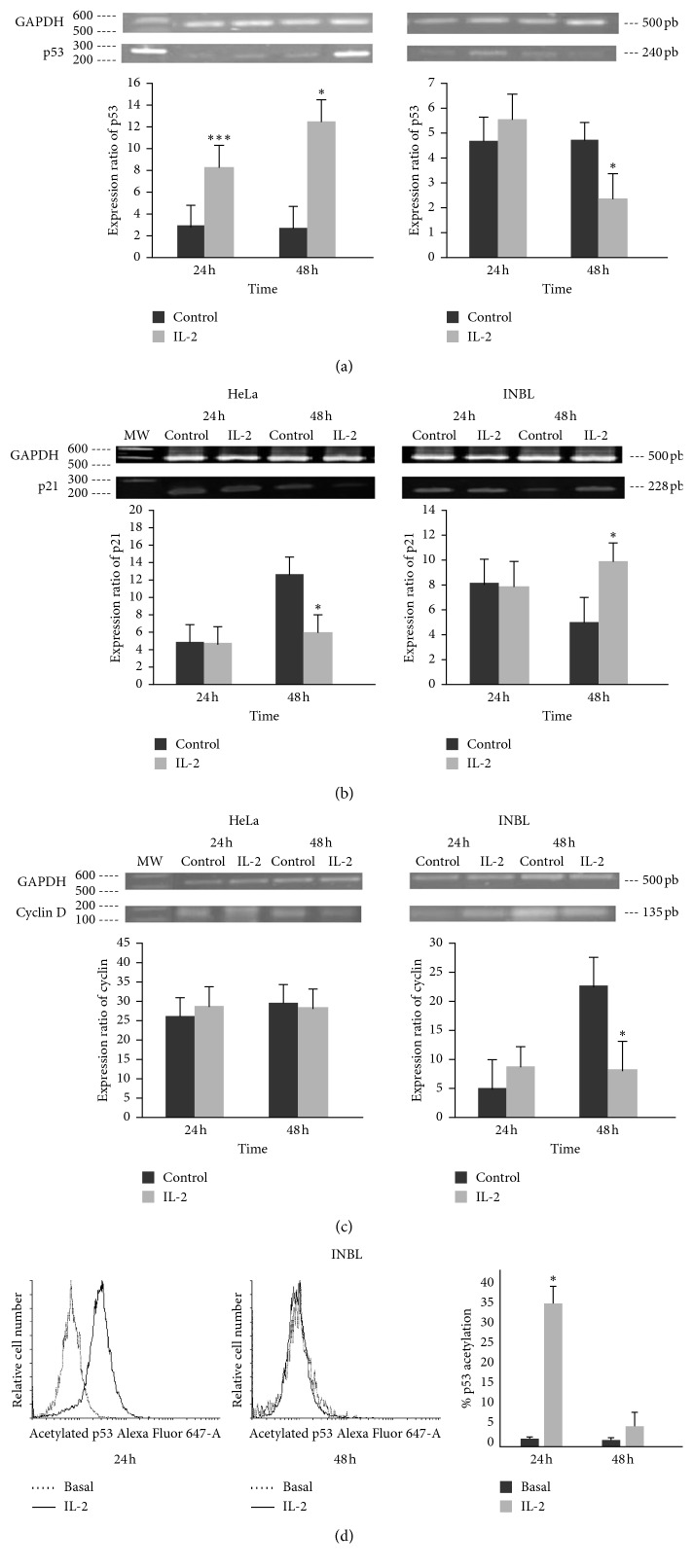
Effect of IL-2 on the p53, p21, and cyclin D expression in cervical cancer cells. HeLa and INBL cells were incubated in the presence or absence of 100 IU/ml of IL-2 for 24 and 48 hours. RNA was obtained, RT-PCR was performed, and the products were separated by electrophoresis in an agarose gel. The results show the bands and the expression ratio for p53 (a), p21 (b), and cyclin D (c). (d) Histogram and graph representation of p53 acetylation. Statistical analysis was performed with a Student's *t-*test of paired, nonparametric data. It was performed using the statistical package Graphpad Prism 5.0. ^*∗*^*P* < 0.05.

In INBL cells, p53 mRNA relative expression increases significantly at 24 and 48 hours compared with the control. In HeLa cells, relative expression decreases significantly at 48 hours in comparison to the control (Figure 7(a)). To determine whether IL-2 induced the activation of p53, we measured the acetylated form of p53 by flow cytometry. In untreated INBL cells, the active form of p53 was less than 5%. When cells grew in the presence of 100 IU/mL of IL-2, there was a 90% increase in the acetylation of lysine 382 at 24 hours. At 48 hours of treatment, there was no difference in p53 acetylation (Figure 7(d)).

Once we determined that p53 was activated in response to IL-2, we analysed p21 since this molecule is downstream of p53. The p21 protein regulates the transit from the G1 to S phase by inhibiting the CDK4/6-cyclin D complex. Overexpression of p21 suppresses the proliferation of mammalian cells. Our results showed a significant decrease in p21 relative expression in HeLa cells at 48 hours compared with the control (Figure 7(b)). In INBL cells, there was an increase in p21 relative expression at 48 hours compared with the control (Figure 7(b)). Cyclin D closely relates to cell-cycle arrest in the G1 phase. Therefore, we analysed the relative expression of cyclin D in HeLa and INBL cells. In HeLa cells, there were no differences in cyclin D relative expression compared to the control. In INBL cells, we observed a decrease in cyclin D relative expression compared to the control at 48 hours (Figure 7(c)).

### 3.6. Transient Arrest in the G1 Phase Protects Cervical Cancer Cells from Apoptosis Induced by Cisplatin

Since treatment with IL-2 induced apoptosis in only 10% of the cell population and cell-cycle arrest was transient, we designed an experiment to determine whether IL-2 protects cells from entering apoptosis. For this experiment, we considered the use of cisplatin, a commonly used chemotherapeutic agent that induces apoptosis in cancer cells. A dose-response curve was generated to determine the IC50 values (Supplementary [Supplementary-material supplementary-material-1]). The concentration considered for the subsequent experiments was 5 *μ*g/ml.

**Figure 8:**
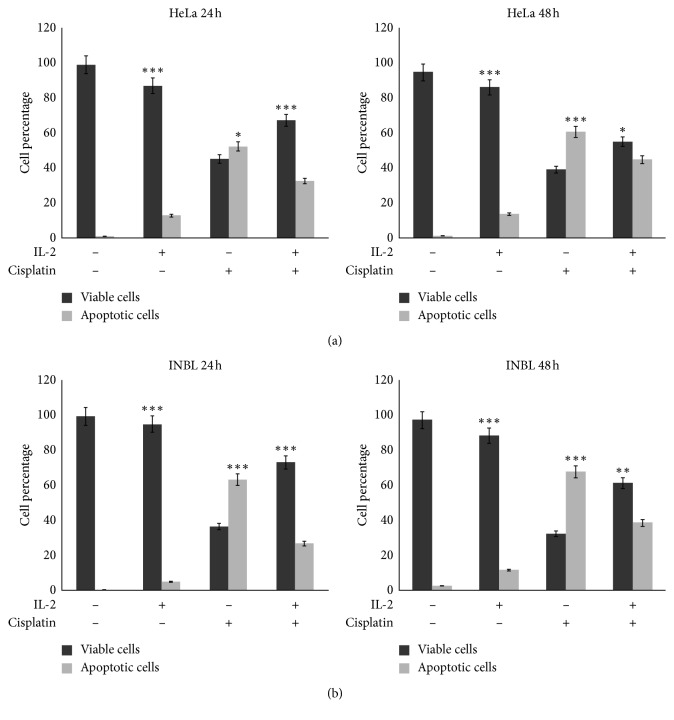
IL-2 protects G1-arrested cervical cancer cells from entering apoptosis. Cells were incubated in the presence or absence of 100 IU/ml of IL-2 for 48 hours. Then, 5 *μ*g/ml of cisplatin was added to the cell cultures, and the mixtures were incubated for 24 and 48 hours. Apoptosis was evaluated using the PE Annexin-V kit apoptosis detection kit. The percentage of apoptotic cells was evaluated by flow cytometry. Percentage of cell death in HeLa cells (a) and INBL cells (b) is shown. Statistical analysis was performed with a Student's *t-*test of paired, nonparametric data. It was performed using the statistical package Graphpad Prism 5.0. ^*∗*^*P* < 0.05, ^*∗∗*^*P* < 0.001, and ^*∗∗∗*^*P* < 0.0001.

Cells were grown in the presence of IL-2 for 48 hours to induce cell-cycle arrest, and cisplatin was added for 24 and 48 hours; untreated cells were used as a control. A definite increase in cell apoptosis was observed with cisplatin treatment in both cell lines. When the cells were arrested with IL-2 and subsequently treated with cisplatin, in the HeLa cell line, there was no difference in the decrease of apoptosis (Figure 8(a)). Surprisingly, in INBL cell line, the percentage of apoptosis was reduced significantly at 24 hours (33.3% to 13.28%) and at 48 hours (37.34% to 20.20%) (Figure 8(b)). The representative cytometry dot plots of the apoptosis assays are shown as supplementary data (Supplementary [Supplementary-material supplementary-material-1]).

Our results showed that after treatment with IL-2, cell proliferation decreased, only 10% of the population died by apoptosis, 59.2% of the cellular population arrested in G1, and the arrested cells offered resistance to death by cisplatin-induced apoptosis. Altogether, our results suggest that treatment with IL-2 does not induce cell death but that this cytokine protects cells from entering apoptosis.

## 4. Discussion

Interleukin-2 plays a major role in the activation and regulation of the immune system, and it is necessary for the maintenance and generation of regulatory T-cells, as well as for the differentiation of helper T-cells in their various subpopulations (for example, Th1, Th2, and Th17 cells). In CD8^+^ T-cells, the IL-2 enhances the generation of effector and memory cells. Thus, IL-2 can enhance the activation of the immune system or its regulation [42]. IL-2 is produced predominantly by activated T helper cells and, to a lesser extent by cytotoxic T lymphocytes, and exhibit a variety of affinity states depending on their receptor subunit composition [43]. IL-2 interacts with three different receptors with different affinities. The low-affinity receptor (*K*_d_∼10^−8^ M) contains only the alpha chain (IL-2R*α*), the receptor with intermediate affinity (*K*_d_∼10^−9^ M) contains the beta and gamma chains (IL-2R*β* and IL-2R*γ*), and the high-affinity receptor (*K*_d_∼10^−11^ M) contains the chains IL-2R*α*, IL-2R*β*, and IL-2R*γ* [12]. The functional receptors are only those of intermediate and high affinity, and the low-affinity receptor is not able to carry out the process of signal transduction [12].

All the above are characteristics found in cells of the immune system; however, the presence of the receptor for IL-2 has also been described in tumour cells and tumour cell lines derived from nonhematopoietic tissue [44]. In this context, our results show the presence of the different subunits of the IL-2 receptor (IL-2R*α*, IL-2R*β*, and IL-2R*γ*) in HPV18^+^ cell lines, derived from cervical tumour tissue (Figure 1) and the response of these tumour cells to the administration of high doses of IL-2 (100 IU/mL). Our data indicate that HeLa and INBL cells decrease their proliferation when treated with high doses of IL-2 for 48 hours (Figure 2). The various effects of IL-2 may be because IL-2 is a cytokine that has a pleiotropic effect. It has been reported that its effects depend on the microenvironment, the concentration, and the type of cell. Also, several studies have shown that IL-2 signals are active in different cellular subpopulations. To date, opposite effects have been reported with the use of IL-2 since low doses of recombinant IL-2 activate Treg cells as an immunosuppressive strategy based on cells against the hyperactive immune system, while high doses of IL-2 have shown success in the stimulation of antitumour immune responses [45–48]. Thus, our results suggest that IL-2 can affect the proliferation of cervical tumour cells in a similar way that it regulates the immune cells.

The decrease in cell proliferation may be due to an increase in cell death, an arrest in the cell cycle or cell senescence, among other cellular processes. It is well established that IL-2 induces cell death in activated T cells; therefore, we decided to evaluate whether the administration of high doses of IL-2 induced cell death by apoptosis. The results show that the decrease in cell proliferation is not due to an increase in cell death by apoptosis; this was measured by analysing the expression of phosphatidylserine (Figure 3) and the loss of symmetry of the plasma membrane, measured with the Annexin/IP cell death kit. Cell death analysis was complemented by evaluating the DNA integrity (Supplementary [Supplementary-material supplementary-material-1]) and the expression of the Bax proapoptotic gene (Figure 4). In all three determinations, we did not find significant differences in comparison to the control, which indicates that there is no cell death due to apoptosis.

After determining that the treatment with high doses of IL-2 induces a decrease in the proliferation in cervical tumour cells and that this decrease does not reflect an increase in the percentage of cell death, we evaluated the phases of the cell cycle. The data show a significant increase in the G1 phase in INBL cells, observed at 48 hours after treatment. It should be mentioned that there was a nonsignificant increase in the G1 phase in HeLa cells. We also observed that when IL-2 is removed, both cell lines reenter the cell cycle to continue their proliferation. These data indicate that the treatment of tumour cells with high doses of IL-2 induces a transient arrest in the cell cycle in the G1 phase. In many cells, an arrest can culminate in senescence or apoptosis, but in most cases, cell arrest is transient. For example, the exposure of fibroblasts to *γ*-radiation promotes a reversible G1 cell-cycle arrest or irreversible senescence-like growth arrest depending on the irradiation dose rate or rate of DNA damage [49]. Our results show that treatment with high doses of IL-2 does not induce cell senescence (Figure 6). It is possible that IL-2 induces a similar process induced by irradiation, but this requires further investigation. Since we observed that high doses of IL-2 induce a transient arrest of the cell cycle in the G1 phase, we analysed if the transcription factor p53 was overexpressed. This factor is highly inducible by various signals such as stress, lack of nutrients [50], DNA damage, and activation of oncogenes [51], among others; it is also important in the arrest of the cell cycle and the induction of apoptosis [52]. Our results show a differential pattern, and HeLa cells do not increase the expression of p53 at 48 hours in response to IL-2. On the contrary, the INBL cells respond to the treatment with IL-2 with an increase in the relative expression of p53 mRNA at 24 and 48 hours (Figure 7(a)). Also, we analysed if IL-2 induced the active form of p53 (acetylated). Our results show that, in the INBL cells, there is an increase in active p53 at 24 hours after the treatment with 100 UI of IL-2 (Figure 7(d)).

Once we observed that cell-cycle arrest correlated with an increase in p53 in INBL cells, we proceeded to evaluate the expression of one of the p53 targets involved in cell-cycle arrest. p21 is under the regulation of p53, and this protein can bind to the complexes Cdk4/cyclin D and Cdk2/cyclin E and inhibits both kinase activities [53]. For this reason, the activation of the p53-p21 pathway is responsible for the response of the phase control point G1, and therefore, we decided to evaluate whether treatment with high doses of IL-2 induces an increase in the expression of p21. Treatment with high doses of IL-2 induced a decrease in p21 expression in HeLa and an increase in INBL cells at 48 hours, suggesting that p21 may be playing an essential role in response to high doses of IL-2 (Figure 7(b)). We also evaluated whether the decrease in proliferation and transient arrest in G1 was due to a reduction in the amount of cyclin D. However, treatment with IL-2 did not affect the expression of cyclin D in HeLa cells, but in INBL cells a decrease in the expression of cyclin D at 48 was observed (Figure 7(c)). The different behaviour of the two cell lines observed in the expression analysis of p53, p21, and cyclin D could be due to the growth pattern of each cell line that is characteristic of the considerable heterogeneity of the tumours that originated cell lines (53). Also, it is possible that like other transcription factors, p53 is able to bind to p53-responsive element (p53RE), and this union may mediate the transactivation of p21 [51], as well as acetylation of p53 that has been shown to be essential for p21 promoter transactivation and cell-cycle arrest in human cancer cell lines [53]. Acetylation of p53 activates transcription through recruitment of coactivators or histone acetyltransferases. So, the increase in p53 expression in INBL cell line at 24 and 48 hours (Figure 7(a)), as well as the increase in p53 activation (Figure 7(d)) at 24 hours, can explain the increase in p21 expression in the INBL cell line and a decrease in the HeLa cell line. Thus, there is a cyclin D decrease, arresting the cell line INBL in G1 phase and nullifying the arrest in HeLa cell line. This difference in the expression of p21 is evident at 48 hours (Figure 7) where there is an increase in INBL cells and a decrease in HeLa cells. p21 is a cyclin-dependent kinase inhibitor and essential negative regulator of proliferation whose transcriptional upregulation by p53 elicits transient cell-cycle arrest.

Our results indicate differential responses in cervical cancer cell lines. These data indicate that not only cells of the immune system respond to cytokines differentially, but tumour cells also have receptors for various cytokines, such as the IL-2 and can respond to high and low doses differentially potentiating or inhibiting its growth. What this sequence of experiments show is how tumour cells respond to high doses of IL-2, and they are transiently arrested probably because of the overexpression and activation of p53, which regulates the expression of p21 and thus cells enter into a G1 arrest.

According to our observations, 100 IU/ml of IL-2 decreased cell proliferation in both cell lines and induced a transient arrest in the G1 phase; however, this transient arrest did not result in cell death. Therefore, we induced cell arrest with IL-2 and treated cells with cisplatin, a well-known inducer of apoptosis. Apoptosis induced by cisplatin in tumour cells generally depends on the p53 pathway. Nonetheless, when tumours possess p53 mutations, cisplatin does not induce apoptosis as effectively, and the clinical effects of this drug are restricted accordingly. Some studies have shown that patients with advanced ovarian cancer harbouring mutations in p53 or overexpressing p53 show low sensitivity to chemotherapy containing cisplatin [54]. Cisplatin is used as a chemotherapeutic agent because it breaks down the double-stranded of DNA-forming adducts [55]. Treatment of human cancer cells with cisplatin promotes arrest in the G2 phase independently of p53 activation [56]. Our results showed that when arrested cells were treated with cisplatin, there was a decrease in apoptosis induced by cisplatin, and an increase in cell viability was observed in INBL cell line (Figure 8). Since IL-2 regulates p21 in cervical cancer cells, one possible explanation for this increase in cell viability is that IL-2 stimulates activation of the DNA repair pathway, in which p21 is an important molecule; p21 binds to and inhibits CDK, preventing the phosphorylation of BRCA, which aids in binding of the nucleoprotein RAD51 to ssDNA, thus promoting DNA repair [57]. It is also possible that p21 co-localizes with nucleotide excision repair (NER) factors that interact with PCNA, is present in complexes containing these NER factors [58], and enhances DNA repair [59]. Our results suggest that cell arrest induced by IL-2 is protecting cells from apoptosis; but further research is necessary to explain this response.

## 5. Conclusion

In summary, HeLa and INBL cells bear the IL-2 receptor, and treatment with a high dose of this cytokine decreases proliferation in both cell lines; however, 10% of cells undergo apoptosis. We also found that treatment with IL-2 did not induce senescence; however, a transient arrest in the G1 phase was observed in INBL cell lines, and p53, p21, and cyclin D appeared to participate in this process. On the other hand, treatment with cisplatin induced apoptosis of the cell population, and when arrested cells were treated with cisplatin, there was a decrease in cells entering apoptosis and an increase in cell survival.

Since cancer cells have active DNA repair capacity, it is possible to speculate that this transient growth inhibition induced by IL-2 facilitates DNA damage repair, enabling cellular recovery, thereby increasing cell viability. Furthermore, it is well known that, in response to DNA damage caused by chemotherapeutic agents such as cisplatin, the activation of the DNA-repair signalling pathway is involved in the resistance of tumour cells to these therapies. Whether IL-2 participates in the resistance of tumour cells by inducing G1 arrest to enable cells to activate DNA repair to increase its survival capacity remains to be addressed in further studies.

## Figures and Tables

**Table 1:** 

Primer	Forward	Reverse	bp
Bax	GTGGTTGGGTGAGACTCCTC	GCAGGGTAGATGAATCGGGG	216
p53	GACACGCTTCCCTGGATTGG	GCTGCCCTGGTAGGTTTTCT	240
Cyclin D1	**GCTGCGAAGTGGAAACCATC**	**CCTCCTTCTGCACACATTTGA**	**135**
p21	**ACTTCCTCCTCCCCACTTGT**	**CACCCTGCCCAACCTTAGAG**	**228**
GAPDH	TTCTTTTGCGTCGCCAGCC	GATGACCCTTTTGGCTCCCC	500

## Data Availability

All data produced during this study are accessible from the corresponding author upon reasonable request.

## Supplementary Materials

Supplementary Materials Supplementary Figure 1: effect of IL-2 on DNA fragmentation in HeLa and INBL cervical cancer cell lines. Supplementary Figure 2: effect of IL-2 on the cell cycle of HeLa and INBL cell lines. Supplementary Figure 3: determination of the mean effective dose of cisplatin (IC50) to induce apoptosis in cervical cancer cells. Supplementary Figure 4: IL-2 protects G1-arrested cervical cancer cells from entering apoptosis.
