# Supplementary material for: IL-2 Induces Transient Arrest in the G1 Phase to Protect Cervical Cancer Cells from Entering Apoptosis
Source: J Oncol. 2019 Sep 26;2019:7475295. doi: 10.1155/2019/7475295 (PMC6791272; doi:10.1155/2019/7475295)
Supplement: Supplementary Materials — Supplementary Figure 1: effect of IL-2 on DNA fragmentation in HeLa and INBL cervical cancer cell lines. Supplementary Figure 2: effect of IL-2 on the cell cycle of HeLa and INBL cell lines. Supplementary Figure 3: determination of the mean effective dose of cisplatin (IC50) to induce apoptosis in cervical cancer cells. Supplementary Figure 4: IL-2 protects G1-arrested cervical cancer cells from entering apoptosis. [file 7475295.f1.pdf]

Supplementary figures

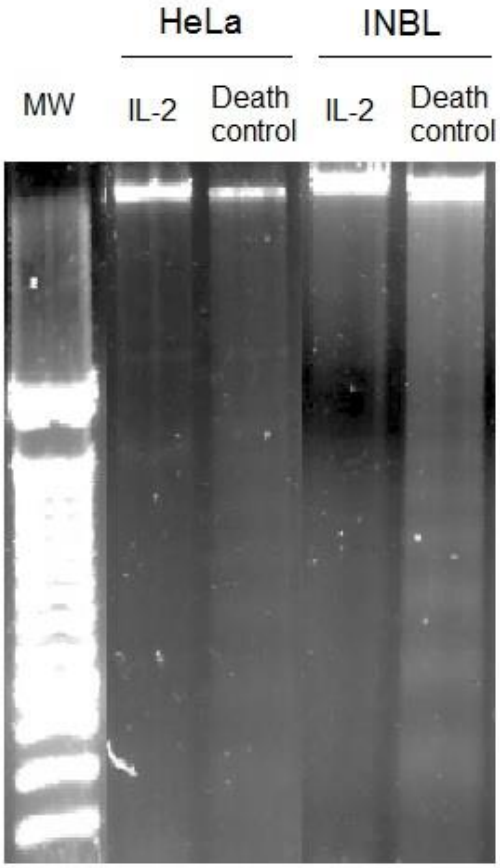

Supplementary figure 1. Effect of IL-2 on DNA fragmentation in HeLa and INBL cervical cancer cell lines. Agarose gel electrophoresis of fragmented cellular DNA induced by puromycin (death control) in comparison with the DNA of cells treated with IL-2. The DNA fragments were visualised by staining with ethidium bromide. MW: Molecular Weight. The gel is a representative image of three independent experiments.

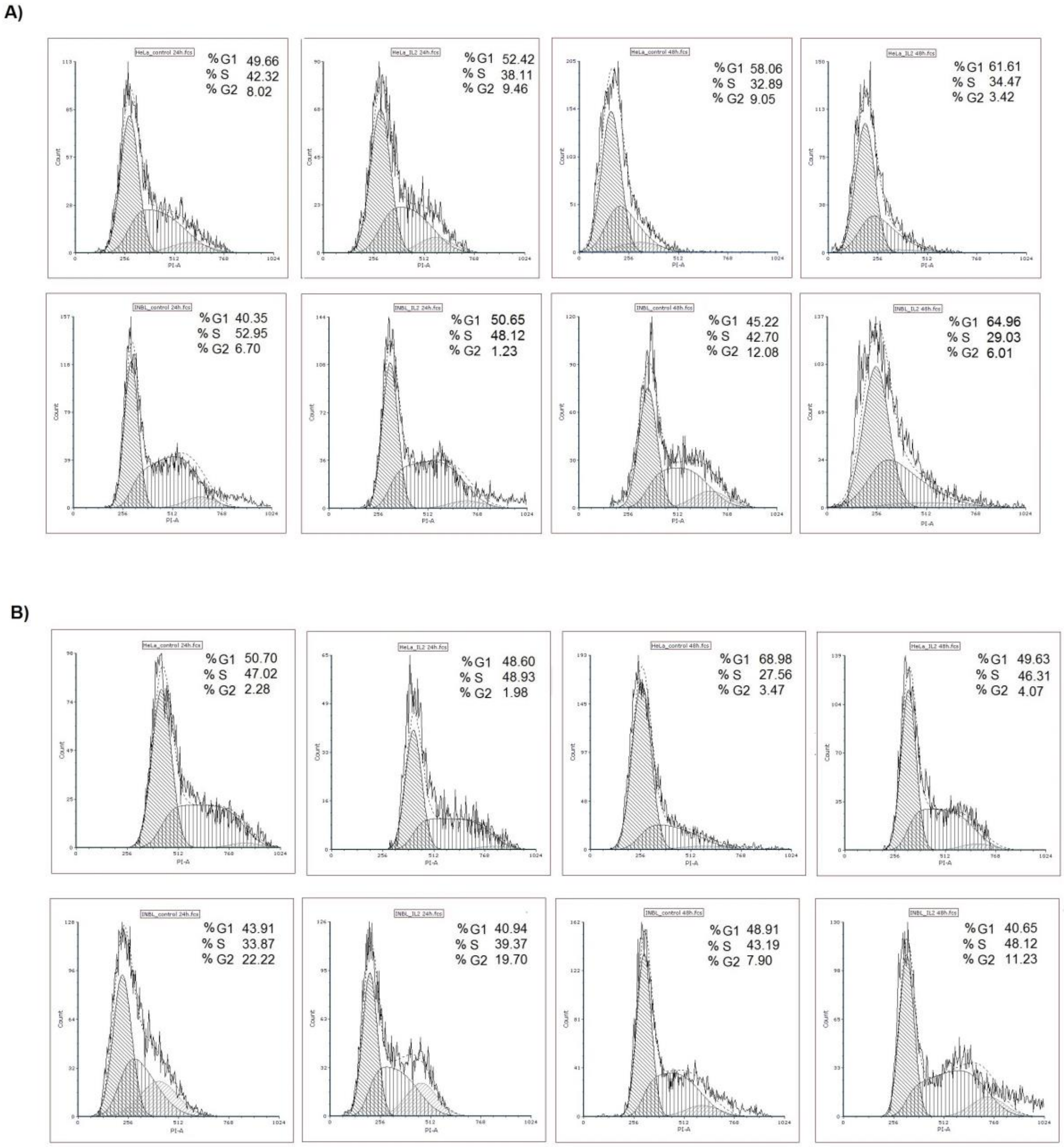

Supplementary figure 2. Effect of IL-2 on the cell cycle of HeLa and INBL cell lines. A) IL-2 induces arrest of the cell cycle in INBL cells at 48 hours. B) After changing the medium for fresh medium free of IL-2, the cells recover their proliferation. Representative images of three independent experiments using flow cytometry on a FACSAria II cytometer (BD, USA).

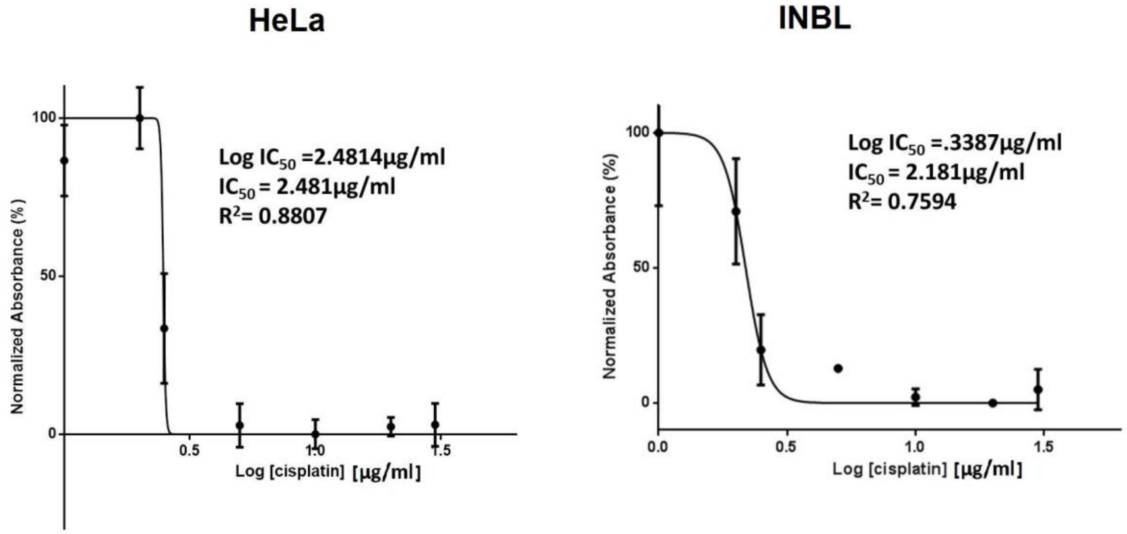

Supplementary figure 3. Determination of the mean effective dose of cisplatin (IC50) to induce apoptosis in cervical cancer cells. To determine the amount of cisplatin required to decrease cell population (IC50), we generated a dose-response curve. A) HeLa and B) INBL cells were incubated with and without 1, 2.5, 5, 10, 20, 30 μg of cisplatin. Statistical analysis was performed using the statistical package Graphpad Prism 5.0.

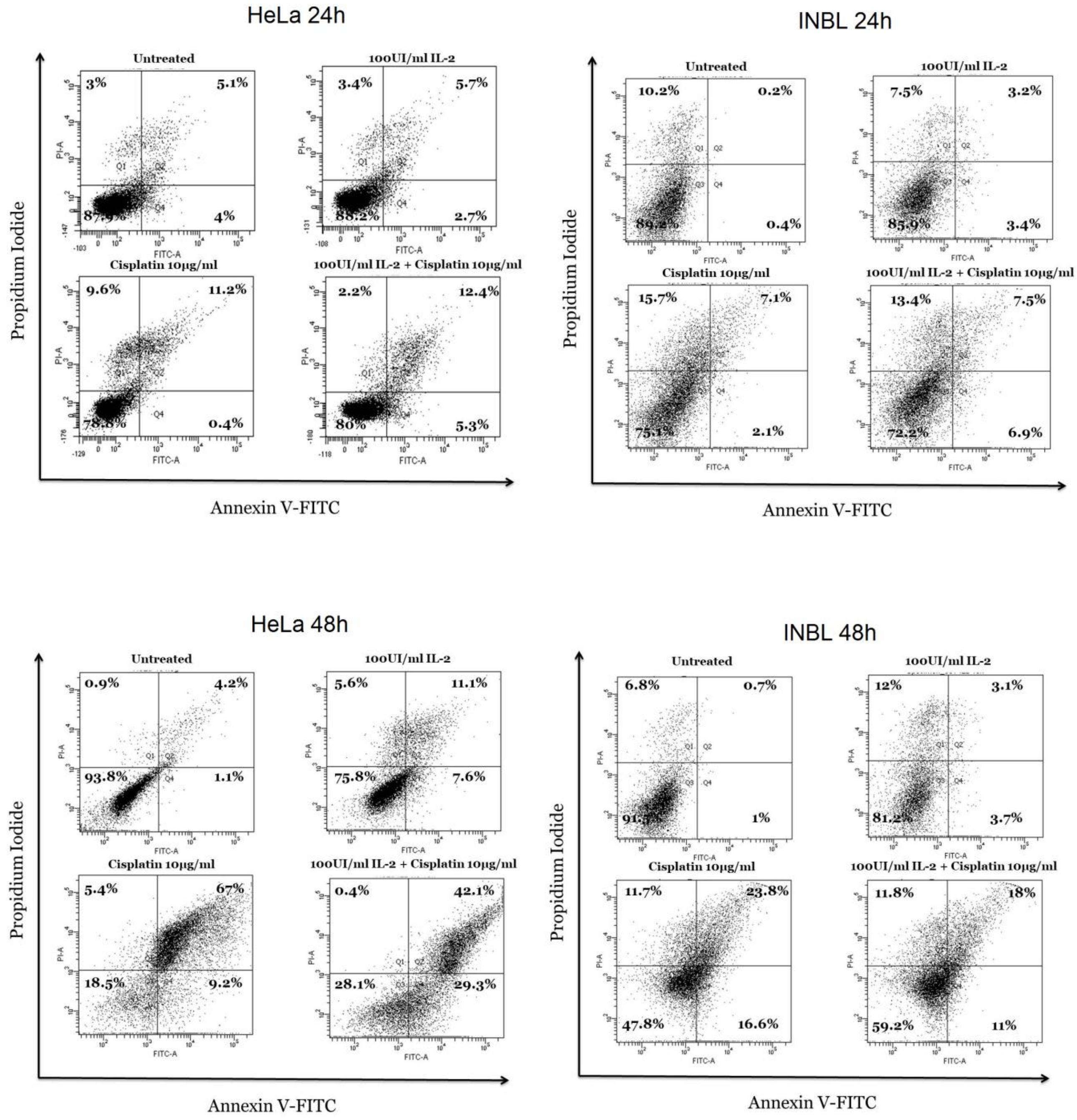

Supplementary figure 4. IL-2 protects G1 arrested cervical cancer cells from entering apoptosis. HeLa and INBL cells were incubated with and without IL-2 for 48 hours. After changing the medium, using fresh medium free of IL-2, cisplatin was added and cells were incubated for 24 hours (A) and 48 hours (B). Dot plots show apoptosis percentage after analysing IP and Annexin V-FITC by flow cytometry. Representative images of one out of three independent experiments are shown.
